# Psychosocial Correlates of Adolescent E-Cigarette Preventive Behavior Among Thai Secondary School Students: A Cross-Sectional Study

**DOI:** 10.3390/healthcare14121664

**Published:** 2026-06-11

**Authors:** Jun Norkaew, Rattanathorn Intarak, Ranee Wongkongdech

**Affiliations:** 1 Faculty of Public Health, Lampang Campus, Thammasat University, Lampang 52000, Thailand; jun.n@fph.tu.ac.th; 2 Faculty of Public Health, St Teresa International University, Nakhon Nayok 26000, Thailand; rattanathorn@trsu.ac.th; 3 Faculty of Medicine, Mahasarakham University, Mueang Maha Sarakham, Maha Sarakham 44000, Thailand

**Keywords:** e-cigarettes, adolescents, preventive behaviors, family attachment, peer influence, confirmatory factor analysis, factor-score regression, indirect association analysis, Thailand

## Abstract

**Highlights:**

**What are the main findings?**

- Family attachment is the strongest psychosocial correlate of adolescent e-cigarette preventive behavior.
- Peer influence exerts a stronger indirect role than attitudes in shaping preventive pathways.

**What are the implications of the main findings?**

- Family- and peer-oriented strategies may complement regulatory approaches in adolescent e-cigarette prevention.
- Strengthening parental engagement and peer environments may enhance prevention effectiveness.

**Abstract:**

**Background:** The increasing use of e-cigarettes among adolescents is a growing public health concern in Thailand, where they are prohibited but remain accessible. This study aimed to examine the psychosocial correlates of preventive behaviors regarding e-cigarettes among adolescents in central Thailand. **Methods:** A cross-sectional correlational study was conducted with 383 secondary school students (Grades 7–12) selected through proportionate stratified random sampling from two government schools in Ongkharak District, Thailand. Data were collected using a validated self-administered online questionnaire assessing attitudes toward e-cigarettes, peer influence, family attachment, and preventive behaviors. Item analysis, confirmatory factor analysis (CFA), and factor-score regression with bootstrapped indirect-association analysis (k = 5000) were performed to examine direct and indirect associations. **Results:** The four-factor measurement model demonstrated acceptable absolute fit (SRMR = 0.069) but weaker incremental fit (CFI = 0.70), expected given the large number of ordinal indicators estimated via maximum likelihood, with standardized factor loadings ranging from 0.621 to 0.926 (*p* < 0.001). The structural model explained 44.2% of the variance in preventive behaviors (R^2^ = 0.442). Family attachment showed the strongest total association (β = 0.456), including both direct and indirect associations through attitudes (β = 0.116) and peer influence (β = 0.162), consistent with a pattern of statistically significant indirect associations. **Conclusions:** Family attachment was associated with self-reported e-cigarette preventive behavior, with statistically significant indirect associations through attitudes and peer influence. Given the cross-sectional design, these findings should be interpreted as model-consistent associations rather than causal mediation, and may inform future family- and peer-oriented prevention research in comparable settings.

## 1. Introduction

Electronic cigarettes (e-cigarettes) have emerged as a major public health concern among adolescents worldwide. Data from the Global Youth Tobacco Survey indicate that e-cigarette use has increased in more than 30 countries, in some cases surpassing conventional cigarette smoking among adolescents aged 13–15 years [1]. In Thailand, despite strict legal prohibitions on the import and sale of e-cigarettes under the Non-Smokers’ Health Protection Act B.E. 2535, national surveillance data report a prevalence of approximately 9.1% among secondary school students, with initiation occurring as early as 13 years of age [2].

E-cigarettes expose users to nicotine, formaldehyde, acetone, and other harmful substances that can adversely affect adolescent brain development and respiratory health [3,4]. Adolescents are particularly vulnerable to marketing influences and peer-driven initiation, with evidence indicating that most Thai youth who use e-cigarettes are introduced to them by friends [2]. Despite these risks, the psychosocial mechanisms that may protect adolescents from initiating e-cigarette use remain insufficiently understood, particularly in the Thai context.

Preventive behaviors—such as refusing offers, avoiding high-risk environments, seeking accurate health information, and demonstrating media literacy—represent proximal protective factors against substance use initiation. These behaviors are influenced by individual attitudes, peer norms, and family relationships, as conceptualized in the Theory of Reasoned Action (TRA) [5] and the Theory of Planned Behavior (TPB) [6]. The present study draws selectively on these frameworks, focusing on attitudes and peer-related social norms as correlates of preventive behavior, while acknowledging that key TRA/TPB constructs such as behavioral intention and perceived behavioral control were not measured. Family attachment, while not a core TRA/TPB construct, is incorporated as a family-context protective factor theorized to relate to both attitudes and the peer environments adolescents engage with.

Family attachment, defined as the degree of emotional closeness, care, and relational satisfaction within the family unit, has consistently been identified as a key protective factor against adolescent risk behaviors in longitudinal research [7]. Strong family relationships may reinforce negative attitudes toward substance use and influence the peer environments adolescents engage with, thereby shaping social norms [8]. However, the extent to which family attachment is associated with preventive behaviors indirectly through attitudes and peer influence—consistent with a mediation pattern—has not been empirically examined in the Thai secondary school context.

Previous studies in Thailand [9,10] and internationally [8,11,12] have primarily focused on bivariate relationships using regression-based approaches, which are limited in their ability to assess indirect pathways and account for measurement error in latent constructs. Structural equation modeling (SEM) provides a more comprehensive analytical framework by simultaneously estimating measurement and structural models, allowing for the examination of both direct and indirect associations with greater statistical rigor.

It is important to note that this study employs a cross-sectional design. Accordingly, the mediation analysis does not establish causal relationships but rather identifies patterns of association consistent with hypothesized pathways. This limitation is explicitly acknowledged, and findings are interpreted with appropriate caution.

Therefore, this study aimed to: (1) examine the measurement properties of a four-construct model using confirmatory factor analysis (CFA); (2) estimate the direct and indirect associations among family attachment, attitudes, peer influence, and preventive behaviors; and (3) evaluate whether attitudes and peer influence statistically mediate the relationship between family attachment and preventive behaviors among secondary school students in Thailand.

## 2. Materials and Methods

### 2.1. Study Design and Setting

A cross-sectional study with correlational design was conducted during the 2025 academic year in Ongkharak District, Nakhon Nayok Province, Thailand. This peri-urban district is considered broadly representative of central Thai settings.

### 2.2. Participants and Sampling

The target population comprised 1984 students enrolled in Grades 7–12 (Mathayom 1–6) across two government secondary schools. A minimum sample size of 383 participants was calculated using Yamane’s formula with a 95% confidence level and a margin of error of 0.05 [13]. Participants were selected using proportionate stratified random sampling across grade levels, followed by simple random sampling within each stratum. Inclusion criteria were: (1) currently enrolled students, (2) aged 12–18 years, and (3) willingness to participate. Exclusion criteria included absence on the day of data collection and incomplete questionnaire responses (>10% missing data).

Of the 1984 eligible students, 383 were selected through proportionate stratified random sampling and invited to participate. All 383 students provided consent and completed the online questionnaire. One participant had missing responses on seven items (P21–P27; 1.2% of items), which fell below the 10% exclusion threshold; item-level missing data for this participant were handled using item-mean imputation within the relevant subscale. The final analytical sample therefore comprised 383 participants.

### 2.3. Instruments

Data were collected using a structured, self-administered online questionnaire (Google Forms) comprising five sections. All items were measured on a five-point Likert scale (1 = strongly disagree/never to 5 = strongly agree/always).

Preventive behaviors toward e-cigarette use (Section 5) were assessed using 27 items adapted from Hatti and Phongnantakulkit [10], covering five domains: risk avoidance, refusal skills, health information seeking, media literacy, and family-supported avoidance. Attitudes toward e-cigarette use (Section 2) included 14 items assessing cognitive and affective evaluations. Peer influence (Section 3) comprised 11 items measuring peer modeling and reinforcement of health-protective behaviors. Family attachment (Section 4) included 12 items assessing emotional closeness, communication, care, and relational satisfaction within the family.

Content validity was established by three public health experts, yielding Content Validity Index (CVI) values ranging from 0.82 to 0.92. Internal consistency reliability (Cronbach’s α) ranged from 0.85 to 0.92 across subscales in a pilot study. All instruments are provided in the Supplementary Materials.

### 2.4. Data Analysis

Descriptive statistics and Pearson’s correlation coefficients were computed using IBM SPSS Statistics (version 26). Structural analyses were conducted using Python (version 3.11) with NumPy and SciPy libraries. Statistical significance was set at *p* < 0.05.

To assess the potential for common method bias arising from the single-source, single-occasion design, Harman’s single-factor test was conducted on all 58 retained items. An exploratory principal component analysis was performed without rotation, and the variance explained by the first unrotated factor was examined. The first factor accounted for 43.1% of the total variance (eigenvalue = 24.996), which is below the commonly applied threshold of 50%. The presence of four additional factors each explaining more than 2.8% of variance further indicates that the data are not dominated by a single method factor. These results suggest that common method bias is unlikely to pose a serious threat to the validity of the present findings, though this assessment should be interpreted with caution given the limitations of Harman’s test.

Item analysis was performed using corrected item–total correlations (CITC ≥ 0.30) and alpha-if-item-deleted criteria. Based on this analysis, Attitudes was reduced from 14 to 13 items (α = 0.942), Family Attachment from 12 to 11 items (α = 0.964), and Preventive Behavior from 27 to 23 items (α = 0.981), while all Peer Influence items were retained (α = 0.911).

A two-step analytic approach comprising CFA followed by factor-score regression was employed [14]. First, confirmatory factor analysis (CFA) was conducted to evaluate the measurement model. Model fit was assessed primarily using the standardized root mean square residual (SRMR ≤ 0.08), alongside the comparative fit index (CFI) and Tucker–Lewis index (TLI). Convergent validity was evaluated using average variance extracted (AVE ≥ 0.50) and composite reliability (CR ≥ 0.70) [15], while discriminant validity was assessed using the heterotrait–monotrait ratio (HTMT < 0.85) [16]. Given the large number of indicators (*p* = 58) and the ordinal nature of Likert-scale data, chi-square-based indices (CFI and TLI) are known to be downwardly biased; therefore, SRMR was treated as the primary model fit criterion [17].

In the second step, the structural model was estimated using factor score regression with bootstrapped standard errors. Indirect effects were evaluated using bias-corrected accelerated (BCa) bootstrapping with 5000 resamples [18]. Indirect effects were considered statistically significant when the 95% BCa confidence interval did not include zero. Given the cross-sectional design, these findings are interpreted as patterns of association consistent with mediation rather than evidence of causal relationships. The proportion of the total association attributable to indirect pathways was calculated as the ratio of the total indirect effect to the total effect.

### 2.5. Ethical Considerations

This study was approved by the Human Research Ethics Committee of St Teresa International University, Thailand (Approval No. TRSU014/2568; approved on 5 April 2025). The study was conducted in accordance with the Declaration of Helsinki (1975, revised in 2013).

Informed consent was obtained from all participants and their legal guardians prior to data collection. Participation was voluntary, and confidentiality and anonymity were strictly maintained throughout the study.

## 3. Results

### 3.1. Participant Characteristics

A total of 383 secondary school students participated in the study (Table 1). The majority were female (n = 223, 58.2%). Most participants were aged 14–15 years (n = 163, 42.6%), with the largest proportion enrolled in Grade 9 (Mathayom 3) (n = 102, 26.6%). Most students had a cumulative GPA of 3.01–4.00 (n = 231, 60.3%) and reported a monthly family income of 5001–10,000 Thai Baht (n = 143, 37.3%).

### 3.2. Descriptive Statistics and Bivariate Correlations

Scale-level descriptive statistics and bivariate correlations are presented in Table 2. Attitudes toward e-cigarettes showed the highest mean score (M = 4.338, SD = 0.703), followed by Preventive Behavior (M = 4.109, SD = 0.991), Family Attachment (M = 3.889, SD = 0.940), and Peer Influence (M = 3.763, SD = 0.856). Attitudes and Preventive Behavior exhibited notable negative skewness (−1.436 and −1.351, respectively), indicating ceiling effects consistent with the generally high endorsement of negative e-cigarette attitudes in this sample. One participant had missing data on seven Preventive Behavior items (P21–P27), which were handled using item-mean imputation within the subscale.

All bivariate correlations among the four constructs were statistically significant (*p* < 0.001). Preventive Behavior showed the strongest correlation with Peer Influence (r = 0.566), followed by Attitudes (r = 0.521) and Family Attachment (r = 0.457). Family Attachment was also moderately correlated with both Peer Influence (r = 0.458) and Attitudes (r = 0.372). None of the intercorrelations exceeded 0.60, providing preliminary support for discriminant validity.

### 3.3. Measurement Model (CFA)

Following item analysis, confirmatory factor analysis (CFA) was conducted on the refined four-factor model comprising 58 indicators (13 + 11 + 11 + 23). The measurement model showed acceptable absolute fit based on SRMR (0.069), but weak incremental fit based on CFI (0.70); therefore, the structural associations should be interpreted cautiously. The chi-square statistic was significant [χ^2^ (1593) = 9130.51, *p* < 0.001], consistent with the known sensitivity of this index to large sample sizes and model complexity [17].

All standardized factor loadings were statistically significant (*p* < 0.001), ranging from 0.621 to 0.926 (Table 3). Convergent validity was supported for all constructs, with composite reliability (CR) values ranging from 0.930 to 0.983 and average variance extracted (AVE) values exceeding 0.50.

Discriminant validity was also supported, with all heterotrait–monotrait ratio (HTMT) values below 0.85 (range: 0.392–0.595) and AVE exceeding the maximum shared variance (MSV) for each construct (Table 3 and Table 4).

### 3.4. Structural Model and Indirect Associations

The structural model showed acceptable absolute fit (SRMR = 0.069) with weak incremental fit (CFI = 0.70) and explained 44.2% of the variance in preventive behavior (R^2^ = 0.442), as well as 21.2% of the variance in peer influence and 13.9% of the variance in attitudes. All hypothesized direct paths were statistically significant (*p* < 0.001; Table 5).

Family attachment was positively associated with both attitudes (β = 0.373, 95% CI [0.263, 0.486]) and peer influence (β = 0.460, 95% CI [0.372, 0.553]). Both peer influence (β = 0.353, 95% CI [0.239, 0.471]) and attitudes (β = 0.309, 95% CI [0.203, 0.438]) were significantly associated with preventive behavior. The direct association between family attachment and preventive behavior remained significant after accounting for both variables (β = 0.178, 95% CI [0.066, 0.291]).

Bootstrapped analyses (k = 5000, BCa) indicated significant indirect associations of family attachment with preventive behavior through attitudes (β = 0.116, 95% CI [0.066, 0.180]) and peer influence (β = 0.162, 95% CI [0.105, 0.235]). The total indirect association was β = 0.278 (95% CI [0.208, 0.364]), and the total association was β = 0.456 (95% CI [0.348, 0.560]).

The pattern of results was consistent with statistically significant indirect associations (consistent with a partial indirect-association pattern), with 60.9% of the total association accounted for by indirect pathways.

### 3.5. Covariate-Adjusted Sensitivity Analysis

To address reviewer concerns regarding the absence of covariate adjustment, three supplementary OLS regression models were estimated controlling for sex, grade level, GPA, and family income. All 383 participants provided complete data on demographic covariates. Results are summarized in Table 6.

In Model 1 (Attitudes~Family Attachment + covariates; R^2^ = 0.200), Family Attachment remained a significant correlate (β = 0.350, *p* < 0.001) after controlling for all demographic variables. GPA was the only significant covariate (β = 0.238, *p* < 0.001); sex, grade level, and income were not significant. In Model 2 (Peer Influence~Family Attachment + covariates; R^2^ = 0.308), Family Attachment again showed a robust association (β = 0.380, *p* < 0.001), with grade level (β = 0.163, *p* < 0.001) and GPA (β = 0.223, *p* < 0.001) also significant. In Model 3 (Preventive Behavior~Family Attachment + Attitudes + Peer Influence + covariates; R^2^ = 0.454), all three psychosocial predictors remained significant: Family Attachment (β = 0.192, *p* < 0.001), Attitudes (β = 0.289, *p* < 0.001), and Peer Influence (β = 0.341, *p* < 0.001). GPA was the only demographic covariate significantly associated with preventive behavior (β = 0.093, *p* = 0.037). The R^2^ for the outcome model increased marginally from 0.442 (unadjusted) to 0.454 (adjusted), indicating that demographic variables account for minimal additional variance beyond the psychosocial predictors. The direction and relative magnitude of all psychosocial associations were consistent with the unadjusted model, supporting the robustness of the main findings.

## 4. Discussion

This study examined the psychosocial correlates underlying e-cigarette preventive behaviors among Thai secondary school students using CFA-informed factor-score regression with bootstrapped indirect-association analysis. The findings suggest that family attachment was the strongest psychosocial correlate, with statistically significant associations—both direct and indirect—through attitudes and peer influence. These patterns are consistent with, but do not establish, the mediation pathways proposed by the Theory of Reasoned Action (TRA) and the Theory of Planned Behavior (TPB). Given the cross-sectional design, these findings reflect associations rather than causal mechanisms.

The finding that family attachment was the strongest correlate is consistent with longitudinal evidence. Resnick et al. [7] identified family connectedness as a robust protective factor against adolescent risk behaviors across diverse populations. In the Thai context, familial relationships are embedded in a collectivist cultural framework in which parent–child bonds and family norms play central roles. The observation that a substantial proportion of the association between family attachment and preventive behavior remained direct suggests that factors such as parental monitoring, rule-setting, and family-based behavioral regulation may also contribute, although longitudinal research is required to clarify these mechanisms.

The indirect pathway through the protective peer environment construct (β = 0.162) was slightly stronger than that through attitudes (β = 0.116), despite attitudes being a primary focus of many prevention programs. It should be noted that the peer influence items in this study primarily assessed a prosocial and protective peer environment (e.g., friends encouraging exercise, constructive activities, and emotional support) rather than direct peer pressure or e-cigarette-specific norms. Accordingly, the construct may be more precisely characterized as a “protective peer environment,” and findings should be interpreted in that light. This finding aligns with social learning theory, which emphasizes the role of observational learning in shaping behavior [19], and is consistent with evidence from Korean [20] and Australian cohorts [21], which identify peer context as a proximal correlate of adolescent behavior. The stronger association between family attachment and the protective peer environment (β = 0.460) than between family attachment and attitudes (β = 0.373) suggests that family relationships may be linked to the peers adolescents engage with and the norms they internalize. These findings have practical implications, suggesting that family-strengthening interventions that support parental engagement in shaping peer contexts may be particularly relevant [11].

Attitudes toward e-cigarette use remained a significant correlate (β = 0.309) and indirect pathway (β = 0.116), consistent with TRA [5] and prior Thai studies [9,10]. The high mean attitude score (M = 4.338) suggests that most students already hold negative views toward e-cigarettes, which aligns with the high level of preventive behaviors observed (M = 4.109). However, lower scores in media literacy indicate potential vulnerability to digital marketing, consistent with international evidence on adolescent exposure to social media promotion of e-cigarettes [3].

The structural model explained 44.2% of the variance in preventive behavior, which is comparable to findings from SEM-based studies in similar populations [8,12]. The observed pattern, consistent with a pattern of statistically significant indirect associations, provides a more integrated account than regression-based approaches by illustrating the interrelation between distal and proximal factors.

These findings may also be relevant to other low- and middle-income countries facing similar gaps between regulatory enforcement and adolescent risk behaviors, where family- and peer-focused strategies may complement existing policy measures.

### 4.1. Policy Implications

These findings have potential implications for future prevention research and program development for Thailand’s e-cigarette prevention framework. The observed prevalence of approximately 9.1% among secondary school students [2], despite legal prohibition, suggests that supply-side enforcement alone may be insufficient. The structural associations identified in this study indicate that demand-side psychosocial strategies—particularly those addressing family attachment and peer norm formation—may serve as important complements to existing regulatory approaches [12].

At the school level, health education curricula may benefit from extending beyond knowledge- and attitude-based components to include structured peer mentoring and family engagement strategies. Family-strengthening approaches—such as parental monitoring, open communication, and guidance on peer group interactions—have been shown to support protective behaviors and could be incorporated into school-based health promotion programs.

At the national level, agencies such as the Thai Food and Drug Administration and the Department of Disease Control may consider incorporating family-referenced social norms into public communication strategies, rather than relying solely on individual-level health messaging. The high endorsement of family-related attitudinal items in this study suggests that such approaches may be particularly relevant in the Thai cultural context.

In addition, regulatory bodies such as the National Broadcasting and Telecommunications Commission may play a role in strengthening oversight of e-cigarette-related content on digital platforms, particularly in light of the observed gaps in media literacy-related preventive behaviors. Together, these findings highlight the potential value of integrated policy approaches that combine regulatory enforcement with family- and peer-oriented interventions.

### 4.2. Limitations

Several limitations should be acknowledged. First, the cross-sectional design precludes causal inference; the mediation findings reported here reflect patterns of association rather than causal mechanisms, and temporal ordering among constructs cannot be established. Longitudinal studies are needed to clarify the directionality of these relationships.

Second, the sample was drawn from two schools within a single district in Nakhon Nayok Province, which may limit the generalizability of the findings to other regions or school contexts.

Third, the use of self-reported measures introduces the possibility of social desirability bias, particularly for items related to adherence to family and school norms.

Fourth, this study did not include measures of actual e-cigarette use behavior. Future research incorporating behavioral outcomes would allow direct comparison between users and non-users and provide a more comprehensive understanding of preventive pathways.

Finally, the relatively large number of indicators in the measurement model may have contributed to biased chi-square-based fit indices under maximum likelihood estimation. Future studies may consider alternative estimators more appropriate for ordinal Likert-scale data.

Additional limitations include the following: (a) the structural model was estimated using factor-score regression following CFA rather than a fully integrated latent-variable SEM, which does not preserve all advantages of simultaneous latent modeling; (b) the global measurement model showed poor incremental fit (CFI = 0.70), which should be interpreted with caution despite acceptable SRMR; (c) the preventive behavior construct may be multidimensional, and its family-related items may partially overlap with the family attachment predictor, potentially inflating observed associations; (d) school- and classroom-level clustering was not accounted for in the analysis, which may have resulted in underestimated standard errors; (e) covariate adjustment was conducted as a sensitivity analysis for demographic variables such as sex, age, grade, GPA, or family income; (f) common method bias was assessed using Harman’s single-factor test, but this approach is limited; future studies should use stronger procedural or statistical controls; and (g) the high internal consistency coefficients (α = 0.982 for preventive behavior) may reflect item redundancy rather than psychometric precision.

## 5. Conclusions

Family attachment was the strongest psychosocial correlate of adolescent e-cigarette preventive behaviors in this cross-sectional school-based sample, with statistically significant associations through attitudes and a protective peer environment. These patterns are consistent with a hypothesized indirect-association model rather than established causal mediation.

In contexts where regulatory enforcement alone may be insufficient, these results highlight the potential value of family- and peer-oriented strategies as complements to existing tobacco control measures.

Given the cross-sectional design, the observed relationships should be interpreted as patterns of association rather than causal pathways. Future longitudinal research is needed to clarify the directionality and mechanisms underlying these relationships.

## Figures and Tables

**Table 1:** Participant characteristics (n = 383).

Characteristic	n	%
**Gender**		
Male	160	41.8
Female	223	58.2
**Educational level** (Grade/Mathayom)		
Grade 7 (M.1)	23	6.0
Grade 8 (M.2)	95	24.8
Grade 9 (M.3)	102	26.6
Grade 10 (M.4)	62	16.2
Grade 11 (M.5)	51	13.3
Grade 12 (M.6)	50	13.1
**Cumulative GPA** (GPAX)		
<2.00	14	3.7
2.01–3.00	138	36.0
3.01–4.00	231	60.3
**Monthly family income** (THB)		
<5000	24	6.3
5001–10,000	143	37.3
10,001–20,000	142	37.1
>20,000	74	19.3

**Note:** GPA = grade point average; THB = Thai Baht.

**Table 2:** Descriptive statistics and bivariate correlations among study constructs (n = 383).

Construct	M	SD	Skew	Kurt	1	2	3	4
1. Attitudes	4.338	0.703	−1.436	2.581	—			
2. Peer Influence	3.763	0.856	−0.672	0.247	0.416 ***	—		
3. Family Attachment	3.889	0.940	−0.640	−0.232	0.372 ***	0.458 ***	—	
4. Preventive Behavior	4.109	0.991	−1.351	1.209	0.521 ***	0.566 ***	0.457 ***	—

**Note:** M = mean; SD = standard deviation; Skew = skewness; Kurt = excess kurtosis. Columns 1–4 are Pearson correlation coefficients. *** *p* < 0.001.

**Table 3:** Measurement model results.

Item	Description	λ	λ^2^
Attitudes toward E-Cigarette Use (13 items), α = 0.942, CR = 0.951, AVE = 0.597			
A2	Harmful to health	0.762	0.580
A3	Illegal	0.797	0.636
A4	Contains dangerous addictive substances	0.794	0.630
A5	Negatively affects family relationships	0.827	0.684
A6	Jeopardizes one’s future	0.758	0.575
A7	Associated with other substance use	0.682	0.466
A8	Causes cognitive deterioration	0.726	0.527
A9	Waste of money	0.751	0.564
A10	Highly addictive	0.734	0.539
A11	Damages reputation	0.813	0.660
A12	Negative effects on lungs and heart	0.802	0.643
A13	Causes family distress	0.825	0.681
A14	Often causes social problems	0.764	0.583
Peer Influence (11 items) α = 0.911, CR = 0.930, AVE = 0.550			
GF1	Invites beneficial activities	0.674	0.454
GF2	Provides advice on problems	0.640	0.410
GF3	Warns against e-cigarettes	0.652	0.426
GF4	Invites constructive leisure activities	0.805	0.648
GF5	Encourages academic commitment	0.846	0.716
GF6	Serves as a positive role model	0.800	0.640
GF7	Provides emotional support	0.781	0.610
GF8	Not involved with substances	0.621	0.386
GF9	Encourages physical exercise	0.646	0.418
GF10	Provides academic encouragement	0.806	0.650
GF11	Encourages prosocial activities	0.835	0.697
Family Attachment (11 items) α = 0.964, CR = 0.969, AVE = 0.740			
RFAM2	Family shows care and attention	0.867	0.751
RFAM3	Feels warm when with family	0.880	0.775
RFAM4	Parents provide advice on problems	0.812	0.659
RFAM5	Family spends quality time together	0.846	0.716
RFAM6	Parents understand feelings	0.867	0.751
RFAM7	Satisfied with family relationship	0.864	0.746
RFAM8	Family provides encouragement	0.882	0.778
RFAM9	Feels like an important family member	0.901	0.812
RFAM10	Parents show interest in activities	0.856	0.733
RFAM11	Family shows love and care	0.843	0.711
RFAM12	Happy when with family	0.841	0.708
Preventive Behavior (23 items) α = 0.982, CR = 0.983, AVE = 0.718			
P3–P12	Risk avoidance and refusal skills	0.664–0.884	0.441–0.782
P15–P27	Family-supported and school-based avoidance	0.741–0.926	0.549–0.858

**Note:** Standardized factor loadings, composite reliability, and average variance extracted. λ = standardized factor loading; λ^2^ = communality; CR = composite reliability; AVE = average variance extracted. All factor loadings were statistically significant (*p* < 0.001). Measurement model fit: χ^2^ (1593) = 9130.51, *p* < 0.001. The CFI value below conventional thresholds is expected in models with a large number of indicators and ordinal Likert-scale data estimated using maximum likelihood.

**Table 4:** Convergent and discriminant validity.

Construct	Items	α	CR	AVE	√AVE	MSV	HTMT
Attitudes	13	0.942	0.951	0.597	0.773	0.272	—
Peer Influence	11	0.911	0.930	0.550	0.742	0.320	0.446
Family Attachment	11	0.964	0.969	0.740	0.860	0.210	0.392/0.487
Preventive Behavior	23	0.982	0.983	0.718	0.847	0.320	0.547/0.595

**Note:** Convergent and discriminant validity indices. α = Cronbach’s alpha; CR = composite reliability; AVE = average variance extracted; MSV = maximum shared variance; HTMT = heterotrait–monotrait ratio. All HTMT values were below the threshold of 0.85 (range: 0.392–0.595), and AVE exceeded MSV for all constructs, supporting convergent and discriminant validity.

**Table 5:** Structural model results and indirect associations.

Pathway	β	95% CI	R^2^
**Direct associations**			
Family attachment → Attitudes	0.373	[0.263, 0.486]	0.139
Family attachment → Peer influence	0.460	[0.372, 0.553]	0.212
Family attachment → Preventive behavior	0.178	[0.066, 0.291]	—
Attitudes → Preventive behavior	0.309	[0.203, 0.438]	—
Peer influence → Preventive behavior	0.353	[0.239, 0.471]	0.442
**Pathway**	**β**	**95% CI**	
**Indirect associations (BCa bootstrap, k = 5000)**			
Family → Attitudes → Behavior	0.116	[0.066, 0.180]	
Family → Peer → Behavior	0.162	[0.105, 0.235]	
Total indirect association	0.278	[0.208, 0.364]	
**Pathway**	**β**	**95% CI**	
**Total association**			
Direct association	0.178	[0.066, 0.291]	
Total association	0.456	[0.348, 0.560]	

**Note:** Structural model results and mediation analysis. β = standardized path coefficient; CI = confidence interval; BCa = bias-corrected and accelerated bootstrap. All reported paths were statistically significant (*p* < 0.001). R^2^ for Attitudes = 0.139; Peer Influence = 0.212; Preventive Behavior = 0.442. The proportion of the total association attributable to indirect pathways was 60.9%.

**Table 6:** Covariate-adjusted regression models (n = 383).

Variable	Model 1 Attitudes β	Model 2 Peer β	Model 3 Preventive β	*p* (Model 3)	R^2^
Family Attachment	0.350 ***	0.380 ***	0.192 ***	<0.001	0.200
Attitudes	—	—	0.289 ***	<0.001	
Peer Influence	—	—	0.341 ***	<0.001	
GPA	0.238 ***	0.223 ***	0.093 *	0.037	
Grade level	−0.019	0.163 ***	−0.003	0.941	
Sex (male ref.)	−0.057	−0.033	0.045	0.252	
Family income	−0.078	−0.018	−0.071	0.081	
**R^2^ (full model)**	**0.200**	**0.308**	**0.454**		

**Note:** β = standardized regression coefficient. Model 1 outcome = Attitudes; Model 2 outcome = Peer Influence; Model 3 outcome = Preventive Behavior. All three models adjust for sex, grade level, GPA, and family income simultaneously. * *p* < 0.05; *** *p* < 0.001.

## Data Availability

The datasets generated and/or analyzed during the current study are not publicly available due to ethical and privacy restrictions but are available from the corresponding author on reasonable request.

## Supplementary Materials

The following supporting information can be downloaded at: https://www.mdpi.com/article/10.3390/healthcare14121664/s1, Table S1: STROBE Checklist for Cross-Sectional Studies. Ref. [22] is cited in Supplementary Materials.
