# Supplementary material for: Psychosocial Correlates of Adolescent E-Cigarette Preventive Behavior Among Thai Secondary School Students: A Cross-Sectional Study
Source: Healthcare (Basel). 2026 Jun 11;14(12):1664. doi: 10.3390/healthcare14121664 (PMC13300186; doi:10.3390/healthcare14121664)
Supplement: Supplementary file 1 [file healthcare-14-01664-s001.zip › healthcare-4325143-supplementary.pdf]

SUPPLEMENTARY MATERIAL

STROBE Checklist for Cross-Sectional Studies

Manuscript: Psychosocial Correlates of Adolescent E-Cigarette Preventive Behavior Among Thai Secondary School Students: A Cross-Sectional Study

Note: This checklist follows the STROBE Statement (von Elm et al., 2007; Lancet, 370:1453–1457). Items marked "N/A" are not applicable to this cross-sectional study.

Item No.	Recommendation	Reported	Page / Location
**TITLE AND ABSTRACT**			
1a	Indicate the study's design with a commonly used term in the title or the abstract.	Yes	Title; Abstract (Methods)
1b	Provide in the abstract an informative and balanced summary of what was done and what was found.	Yes	Abstract
**INTRODUCTION**			
2	Background/rationale: Explain the scientific background and rationale for the investigation being reported.	Yes	Introduction, pp. 1–3
3	Objectives: State specific objectives, including any pre-specified hypotheses.	Yes	Introduction, p. 3 (study aims)
**METHODS**			
4	Study design: Present key elements of study design early in the paper.	Yes	Methods, Section 2.1
5	Setting: Describe the setting, locations, and relevant dates, including periods of recruitment, exposure, follow-up, and data collection.	Yes	Methods, Section 2.1–2.2
6	Participants: (a) Give the eligibility criteria, and the sources and methods of selection of participants.	Yes	Methods, Section 2.2
7	Variables: Clearly define all outcomes, exposures, predictors, potential confounders, and effect modifiers. Give diagnostic criteria, if applicable.	Yes	Methods, Section 2.3
8	Data sources/measurement: For each variable of interest, give sources of data and details of methods of assessment (measurement). Describe comparability of assessment methods if there is more than one group.	Yes	Methods, Section 2.3
9	Bias: Describe any efforts to address potential sources of bias.	Yes	Methods, Section 2.4 (Harman's test); Limitations
10	Study size: Explain how the study size was arrived at.	Yes	Methods, Section 2.2 (Yamane's formula, n = 383)
11	Quantitative variables: Explain how quantitative variables were handled in the analyses. If applicable, describe which groupings were chosen and why.	Yes	Methods, Section 2.3–2.4
12a	Statistical methods: Describe all statistical methods, including those used to control for confounding.	Yes	Methods, Section 2.4
12b	Describe any methods used to examine subgroups and interactions.	N/A	—
12c	Explain how missing data were addressed.	Yes	Methods, Section 2.2 (item-mean imputation)

12d	If applicable, describe analytical methods taking account of sampling strategy.	Yes	Methods, Section 2.2 (proportionate stratified random sampling)
12e	Describe any sensitivity analyses.	Yes	Results, Section 3.5 (covariate-adjusted models, Table 5)
**RESULTS**			
13a	Participants: Report numbers of individuals at each stage of study (e.g., numbers potentially eligible, examined for eligibility, confirmed eligible, included in the study, completing follow-up, and analysed).	Yes	Methods, Section 2.2; Results, Section 3.1
13b	Give reasons for non-participation at each stage.	Yes	Methods, Section 2.2
13c	Consider use of a flow diagram.	No	Participant flow described in text; diagram not included (single-stage cross-sectional design)
14a	Descriptive data: Give characteristics of study participants (e.g., demographic, clinical, social) and information on exposures and potential confounders.	Yes	Results, Section 3.1; Table 1
14b	Indicate number of participants with missing data for each variable of interest.	Yes	Results, Section 3.2; Table 2 (footnote)
15	Outcome data: Report numbers of outcome events or summary measures.	Yes	Results, Section 3.2; Table 2 (M, SD, skewness, kurtosis)
16a	Main results: Give unadjusted estimates and, if applicable, confounder-adjusted estimates and their precision (e.g., 95% CI). Make clear which confounders were adjusted for and why they were included.	Yes	Results, Sections 3.4–3.5; Tables 4–5
16b	Report category boundaries when continuous variables were categorized.	N/A	—
16c	If relevant, consider translating estimates of relative risk into absolute risk for a meaningful time period.	N/A	—
17	Other analyses: Report other analyses done—e.g., analyses of subgroups and interactions, and sensitivity analyses.	Yes	Results, Section 3.5 (sensitivity analysis with covariates)
**DISCUSSION**			
18	Key results: Summarise key results with reference to study objectives.	Yes	Discussion, opening paragraph
19	Limitations: Discuss limitations of the study, taking into account sources of potential bias or imprecision. Discuss both direction and magnitude of any potential bias.	Yes	Limitations section (7 points added in revision)
20	Interpretation: Give a cautious overall interpretation of results considering objectives, limitations, multiplicity of analyses, results from similar studies, and other relevant evidence.	Yes	Discussion; Conclusion
21	Generalisability: Discuss the generalisability (external validity) of the study results.	Yes	Limitations; Conclusion
**OTHER INFORMATION**			
22	Funding: Give the source of funding and the role of the funders for the present study and, if applicable, for the original study on which the present article is based.	Yes	Funding section (revised in revision)

STROBE = Strengthening the Reporting of Observational Studies in Epidemiology. N/A = Not applicable.
